# Optimization of Spatial Land Use Patterns with Low Carbon Target: A Case Study of Sanmenxia, China

**DOI:** 10.3390/ijerph192114178

**Published:** 2022-10-30

**Authors:** Li Li, Zhichao Chen, Shidong Wang

**Affiliations:** School of Surveying and Engineering Information, Henan Polytechnic University (HPU), Jiaozuo 454003, China

**Keywords:** land use change, carbon flow, differential evolution algorithm, PLUS, low carbon optimization

## Abstract

Land use change is an important factor in atmospheric carbon emissions. Most of the existing studies focus on modeling the land use pattern for a certain period of time in the future and calculating and analyzing carbon emissions. However, few studies have optimized the spatial pattern of land use from the perspective of the impact of carbon emission constraints on land use structure. Therefore, in this study, the effects of land use change on carbon emissions from 1990 to 2020 were modeled using a carbon flow model for Sanmenxia, Henan, China, as an example. Then, the land use carbon emission function under the low carbon target was constructed, and the differential evolution (DE) algorithm was used to obtain the optimized land use quantity structure. Finally, the PLUS model was used to predict the optimal spatial configuration of land use patterns to minimize carbon emissions. The study produced three major results. (1) From 1990 to 2020, the structural change of land use in Sanmenxia mainly occurred between cultivated land, forest land, grassland and construction land. During this period of land use change, the carbon emissions from construction land first increased and then decreased, but despite the decrease, carbon emissions still exceeded carbon sinks, and the carbon metabolism of land use was still far from equilibrium. (2) Between 2010 and 2020, the area of cultivated land began to decrease, and the area of forest land rapidly increased, and land-use-related carbon emissions showed negative growth. This showed that the structural adjustment of energy consumption in Sanmenxia during the period decreased carbon emissions in comparison with the previous period. (3) A comparison of predicted optimized land use patterns with land use patterns in an as-is development scenario showed a decrease in construction land area of 23.05 km^2^ in 2030 with a steady increase in forest land area and a decrease in total carbon emission of 20.43 t. The newly converted construction land in the optimized land use pattern was concentrated in the ribbon-clustered towns built during urban expansion along the Shaanling basin of the Yellow River and the Mianchi–Yima industrial development area.

## 1. Introduction

Carbon emissions have received widespread attention from countries around the world during recent global warming [1]. Human socio-economic development has resulted in large consumption of natural resources, and global carbon emissions continue to increase [2]. China is the world’s second largest economy and the country’s rapid development has made carbon emissions a key issue. According to China Emission Accounts and Datasets (CEADs), China’s annual carbon emissions are approximately 10 Gt, about a quarter of the world’s total carbon emissions [3], and this figure continues to rise. However, the peak of carbon emissions has not yet been reached despite a series of reductive measures. In September 2020, China proposed a carbon peaking plan at the 75th General Debate of the United Nations General Assembly, intending to peak and achieve a steady decrease in CO2 emissions before 2030. In October 2021, the country issued the Notice of Carbon Peaking Action Plan by 2030. These actions show the great importance that China attaches to the issue of carbon emissions.

Land use change has become an important source of carbon emissions [4,5], and land use carbon emissions are influenced by a combination of several factors, with land use change being particularly important among them. Currently, many studies have focused on carbon emissions from urban construction land [6,7], while land use changes across the region are equally important for carbon emissions. Land use is of great importance in carbon emission reduction. Land is the bearer of natural geographical cover, and land resources support numerous socio-economic activities [8,9]. According to the Fifth Assessment Report of the Intergovernmental Panel on Climate Change (IPCC), land use change accounts for approximately one-third of anthropogenic carbon emissions [10]. In recent years, land use carbon emissions in China have continued to increase [11], and regulation of land use changes has become an important step toward achieving a balance between economic growth and carbon emission reduction, and the accounting of carbon emissions is the basis of scientific analysis of carbon emission reduction [12]. The accurate calculation of carbon emissions is therefore of great importance, but, to date, there is still no uniform standard for doing so. In the Guidelines for National Greenhouse Gas Inventories developed by the IPCC, three carbon emission calculation methods are proposed: the emission factor method, the mass balance method and the actual measurement method [13]. To calculate carbon emissions from land use, most researchers quantify the effect of land use changes on carbon emissions by calculating carbon emission coefficients for different land use types [14,15,16].

Carbon emissions from cultivated land, forest land, grassland, water (rivers and streams, lakes, wetlands) and unused land can be measured directly, but carbon emissions from construction land are measured indirectly through the measurement or calculation of carbon emissions from the energy consumed by human activities on construction land [17]. Zhou et al. [18] estimated land use carbon emissions for the Beijing–Tianjin–Hebei urban agglomeration by calculating the correction factors for energy consumption and basic land use carbon emissions. Zhang et al. [19] measured carbon emissions in the Yellow River Delta for 2000–2019 using land use data and fossil energy consumption data for the same period and determined the spatial and temporal distributions of carbon emissions.

Researchers have studied the effects of carbon emissions from different land use patterns and spatial distributions for periods of time in the past, as well as in the present, and some researchers have turned their attention to land use carbon emissions and their spatial distribution in future periods of time. In modeling and predicting future land use, land use distribution and spatial land use patterns are usually combined. Cunha et al. [20] predicted future changes in land cover for 2033, 2050, 2080 and 2100 in the greater Plata River basin using a CA-Markov model. Tang et al. [21] used CA-Markov and CLUE-S models to predict land use in Changli County, China, in 2028 and to predict the spatial and temporal changes in habitat quality and future development trends constrained by land use in order to provide a scientific basis for regional natural environmental protection and land use planning. Ma et al. [22] used an uncertainty model coupled with a spatial allocation model (GeoSOS-FLUS) to optimize for three different land use scenarios in Wuhan, China, and quantified the inherent uncertainty of land use to determine its optimal spatial distributions. These models were based mainly on historical land use conversion patterns in making predictions and were not capable of optimizing land use patterns for user-specified conditions.

Researchers have begun to optimize land use distribution by varying the objective functions and constraints. Dong et al. [23] combined the GCAM and FLUS models to predict potential land use in China from 2010 to 2100 for SSP and RCP scenarios and found that regional crop changes were sensitive to socio-economic dynamics as well as bioenergy production and that different carbon regimes drove forest change in unique ways. They demonstrated an effective method for predicting regional land use responses to a range of alternative future land use distributions. Han et al. [24] used a multi-objective linear programing model to optimize land use distribution in Shenzhen for two scenarios of varying carbon emission targets and economic development trade-offs for 2020 and 2025. Wang et al. [25] considered the economic and ecological aspects of land use change in constructing an objective function to optimize land resource distribution in the Danjiang basin (Henan section) of China in 2028 using a DE-PSO model.

Many CA models cannot simulate the detailed development of fine-grained land areas for multiple land use types in predicting spatial land use pattern distribution [26]. Liang et al. [26] introduced a patch-generating land use simulation (PLUS) model, which combined a land extension analysis strategy with a CA model based on multiple stochastic patch seeds to simulate the generation and evolution of any multiple types of land patches in a spatio-temporal dynamic manner and to explore the mechanisms of land use change in the simulation process. Xu et al. [27] explained and predicted the expansion of seven different land use types in Hangzhou through the PLUS model, which provides ideas for simulating urban expansion in the future. Wang et al. [28] created a dynamic model of land use change and carbon stock quantification in Boltara, China, under the SSP–RCP scenario through a combination of a system dynamics (SD) model, a patch generation land use simulation (PLUS) model and an InVEST model and found that carbon storage could be increased by controlling economic and population growth, promoting energy transition and expanding forest land use in the study area.

In summary, most existing studies focus on the analysis and quantification of the effects of carbon emissions for different land use patterns as well as modeling and calculation of carbon emissions of land use patterns for a period of time in the future. However, few studies have predicted the future trends of carbon emissions in terms of spatial and temporal patterns of land use or have predicted spatially optimized land use patterns subject to carbon emission constraints. We took the prefecture-level city of Sanmenxia as a case study to illustrate the identification and prediction of carbon emissions from land use patterns and the optimization of land use for low carbon to provide baseline reference for future land resource allocation in Sanmenxia.

## 2. Study Area and Data

### 2.1. Study Area

**Figure 1:**
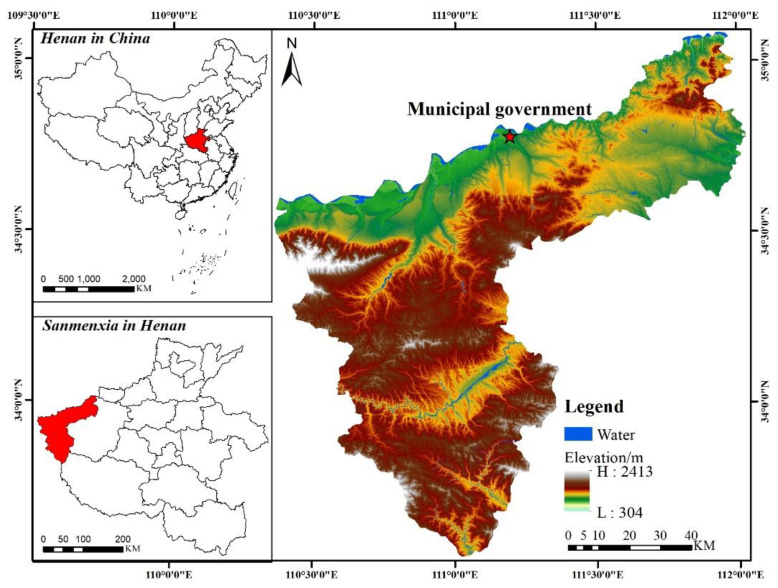
Location of study area.

The prefecture-level city of Sanmenxia, in China, was selected as the study area. It has a strategically important geographical location and is set in a complex topography. Its rapid economic development is typical of a resource-based city, which is now facing serious carbon emission problems. The spatial optimization of future land use patterns under the low carbon aims of the city will provide a foundation for the city’s green development. Sanmenxia is situated in the western part of Henan province where Henan, Shanxi and Shaanxi provinces meet. The landscape is dominated by mountains, hills and the Sichuan plateau. Sanmenxia occupies an area of 9936 km^2^, of which 5421 km^2^ is mountainous, 3250 km^2^ is hilly, and 965 km^2^ is plain. The recorded population was 2,034,872 in 2020, and the GDP was CNY 158.25 billion. Sanmenxia depends on natural resources and has been using land resources unrestrainedly in recent years. Land resources are gradually becoming depleted, and the city is facing several ecological and environmental problems in parallel with economic development challenges, which include increasing air pollution and urban greenhouse effects. Predicting and optimizing future land use patterns with the constraint of low carbon production will provide a foundation for a steady reduction in peak carbon emissions in Sanmenxia and is important for promoting ecologically sound socio-economic practices in Sanmenxia and, indeed, the whole of China. The specific location is shown in Figure 1.

### 2.2. Data

The datasets used in this study include the Raster data, Vector data and Statistical data (Table 1). Land use raster images for 2000, 2010 and 2020 were obtained from the Resource and Environment Science and Data Center of the Chinese Academy of Sciences and were reclassified into six categories of cultivated land, forest land, grassland, water, construction land and bare land, according to the Chinese land use and land cover change (LUCC) system. The slope and slope direction data were obtained using DEM 30 m resolution digital elevation data. The precipitation and temperature data were obtained from the National Meteorological Science Data Sharing Service. Data for the railroads, highways and other roads were obtained from the National Geographic Information Resources Catalogue Service. GDP data for 2019 instead of 2020 were obtained from the Resource and Environment Science and Data Center of the Chinese Academy of Sciences. Data were obtained from the *Statistical Yearbook 2000–2020* for energy consumption and cultivated resources in Sanmenxia and open-source population data.

## 3. Framework

**Figure 2:**
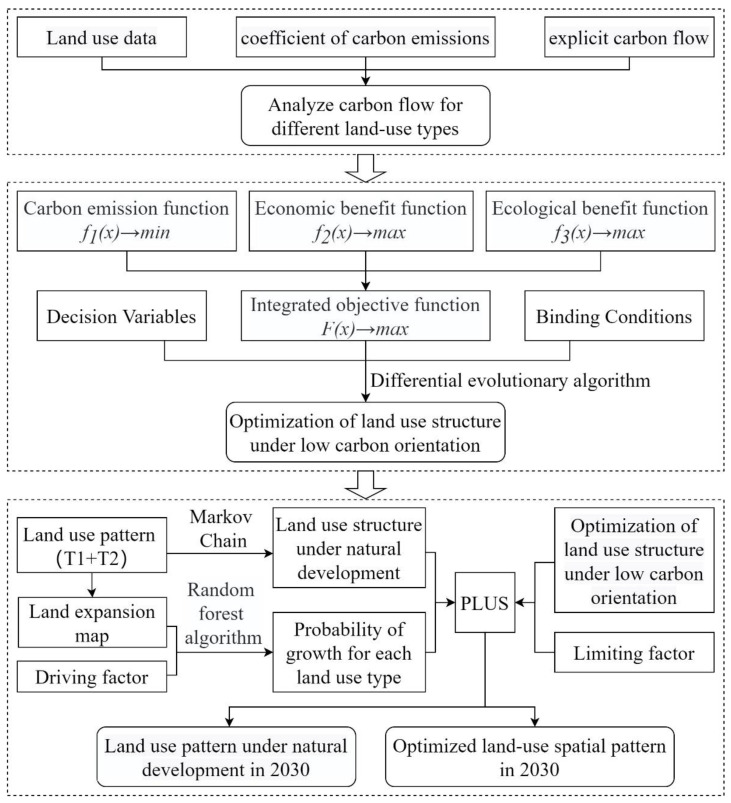
The roadmap of this study.

The intent of this study was to demonstrate the optimization of land use patterns for low carbon production. The optimized land use pattern for low carbon production in 2030 was created by combining a land use carbon emission function, a land use environmental efficiency function and a land use economic efficiency function; the optimal land use pattern structure for 2030 was obtained by using a differential evolutionary algorithm (DE); the optimized land use structure was then fed into a PLUS model to predict the spatial distribution of land use patterns with low carbon production in Sanmenxia in 2030. The technical route of the study is shown in Figure 2.

## 4. Methods

### 4.1. Land Use Carbon Flow Assessment Model

#### 4.1.1. Calculation of Land Use Carbon Emissions

1. Calculation of carbon emissions from construction land

Carbon emissions from construction land were calculated using the quantity of carbon produced by combustion in energy consumption [10,29,30]; default emission factors were used to calculate emissions. Coal, coking coal, crude oil, gasoline, diesel, fuel oil, natural gas and electricity were selected as carbon sources, and total emissions were calculated by

(1) $$C_{j}=\frac{44}{12}\times \sum E_{i}\times f_{i}$$

where $C_{j}$ is the carbon emission of construction land; $E_{i}$ is the consumption of energy source *i*, converted into standard coal for calculation; and $f_{i}$ is the carbon emission coefficient of energy source *i*.

2. Calculation of carbon emissions from other land use types

The carbon emission factors for forest land [31], unused land [32,33], cultivated land [19,34], watersheds [35] and grassland [36] were derived from existing studies and were, respectively, set to −0.664, −0.005, 0.442, −0.253 and −0.021 in units t/ha/y for the target year, calculated as follows.

(2) $$f_{1}(x)=\sum_{i=1}^{6}k_{i}\times S_{i}$$

where $k_{i}$ is the carbon emission factor of all sites in category *i*, and $S_{i}$ is the total land area of sites in category *i*.

#### 4.1.2. Carbon Flow Analysis for Different Land Use Types

An explicit carbon flow model was introduced to better represent the carbon transfer between different land use types and to more accurately assess the effects of land use change on carbon emissions. The model was

(3) $$f_{ij}=(W_{j}-W_{i})\cdot \Delta S$$

(4) $$W_{j}-W_{i}=\frac{V_{j}}{S_{j}}-\frac{V_{i}}{S_{i}}$$

where $f_{ij}$ is the carbon transfer from land use *i* to land use *j*; $W_{j}-W_{i}$ is the change in annual carbon metabolism density during conversion from land use *i* to land use *j*; ΔS is the area affected by the land conversion; and *j* and *i* are the respective carbon fluxes of land use $W_{j}$ and land use $W_{i}$ and are equivalent to the land use type carbon emission factors. $V_{j}$ and $V_{i}$ represent the carbon flow of land type *j* and land type *i*. If $W_{j}-W_{i}>0$, land use conversion is a positive process with an increase in carbon sink, and if $W_{j}-W_{i}<0$, land type transfer is a negative process with a decrease in carbon sink or an increase in carbon emissions.

### 4.2. Low Carbon Optimization Model for Land Use Structure

#### 4.2.1. DE Algorithm to Predict Optimized Land Use Structure

The basic principle of the differential evolution algorithm is to start with a randomly generated initial population and update it iteratively through variation, crossover and selection operations to eliminate unfit individuals and preserve fit individuals and thus approach an optimal solution [37,38]. The basic process of the standard DE algorithm is as follows.

(1) Initialize the population

The basic parameters of the DE algorithm are set. These include the spatial dimension *N*, the population size *NP*, the number of iterations *G*, the variation factor *F*, the crossover factor *CR*, the lower limit of the search space $x_{i,j}^{\min}$ and the upper limit of the search space $x_{i,j}^{\max}$. The initial population is then randomly generated with (*i* = 1, 2,…, *NP*; *j* = 1, 2, …, *N*) and is calculated by

(5) $$x_{i,j}^{0}=x_{i,j}^{\min}+rand(0,1)\times (x_{i,j}^{\max}-x_{i,j}^{\min})$$

where $x_{i,j}^{0}$ is the dimensional component *j* of individual *i* in generation 0, and *rand* (0,1) is a random number uniformly distributed in the interval [0,1].

(2) Variant operation

For each individual $x_{ij}$ (*i* = 1, 2,…, *NP*; *j* = 1, 2, …, *N*) in the population (the target vector), the variant offspring $v_{i}^{\left(g+1\right)}$ is generated by adding the difference between any two individuals in the population to another individual, calculated as follows.

(6) $$v_{i}^{g+1}=x_{r3}^{g}+F(x_{r1}^{g}+x_{r2}^{g})$$

where $v_{i}^{g+1}$ is the generated variance vector; $x_{r1}^{g}$, $x_{r2}^{g}$ and $x_{r3}^{g}$ are three randomly selected individuals in the population and *r*1 ≠ *r*2 ≠ *r*3 ≠ i; *F* is the variance factor, which scales the difference vector $\left(x_{r1}^{g}-x_{r2}^{g}\right)$.

(3) Crossover

To maintain population diversity, the variant offspring $v_{i}^{g+1}$ and the target vector $x_{i}^{g}$ are crossed as follows to produce offspring $u_{i}^{g+1}$ (the test vector):

(7) $$u_{i}^{g+1}=\left\{{}_{x_{ij},rand_{j}>CR\;or\;j\neq j_{rand}}^{v_{i}^{g+1},rand_{j}\leq CR\;or\;j=j_{rand}}\right.$$

where $rand_{j}$ is a random number uniformly distributed in the interval [0,1]; $j_{rand}$ ∈ {1, 2,…, *N*} is a random integer; and *CR* ∈ [0,1] is the crossover factor. In the crossover operation, $j=j_{rand}$ ensures that the test vector $u_{ij}^{g+1}$ differs from the target vector $x_{ij}$ in at least one element, thus maintaining population diversity.

(4) Selection

The basic principle of the selection operation is to compare the target vector with the test vector. If the fitness value of the test vector is greater than the fitness value of the target vector, the target vector is replaced by the test vector in the next generation; otherwise, the target vector is unchanged:

(8) $$x_{i}^{g+1}=\left\{{}_{x_{i}^{g},f(u_{i}^{g+1})>f(x_{i}^{g})}^{u_{i}^{g+1},f(u_{i}^{g+1})\leq f(x_{i}^{g})}\right.$$

where $f(u_{i}^{g+1})$ and $f(x_{i}^{g})$ are the respective fitness values (objective function values) of individuals $u_{i}^{g+1}$ and $x_{i}^{g}$.

#### 4.2.2. The GM(1,1) Model

The GM(1,1) model predicts the carbon emission factor of construction land. It builds a gray system differential prediction model using a small amount of incomplete information to create a fuzzy description of the long-term development of a set of objects and can thus solve problems that are difficult or intractable to resolve using conventional techniques; it has a strong predictive property [39,40]. We developed a gray prediction GM(1,1) model to predict land use carbon emission parameters for 2030. The specific steps of the model are as follows.

(1) Generate the cumulative data sequence $X^{\left(0\right)}=\left\{x^{\left(0\right)}(1),x^{\left(0\right)}(2),\ldots ,x^{\left(0\right)}(N),\right\}$ , which is used to obtain $X^{\left(1\right)}=\left\{x^{\left(1\right)}(1),x^{\left(1\right)}(2),\ldots ,x^{\left(1\right)}(N),\right\}$, where $x^{\left(1\right)}(t)=\sum_{k=1}^{t}x^{\left(0\right)}(k)$.

(2) Solve the parameters by the least square method.

(9) $$a=\left|a\right|={\left(B^{T}B\right)}^{-1}B^{T}Y_{N}$$

where *Y_N_* is the column vector $Y_{N}={\left[x_{1}{}^{\left(0\right)}(2),x_{1}{}^{\left(0\right)}(3),\ldots ,x_{1}{}^{\left(0\right)}(N)\right]}^{T}$, and B is the constructed matrix $B=\left[\begin{array}{cc} -\frac{1}{2}\left(x^{\left(1\right)}(1)+x^{\left(1\right)}(2)\right) & 1\\ -\frac{1}{2}\left(x^{\left(1\right)}(2)+x^{\left(1\right)}(3)\right) & 1\\ \vdots & \vdots \\ -\frac{1}{2}\left(x^{\left(1\right)}(N-1)+x^{\left(1\right)}(N)\right) & 1 \end{array}\right]$

(3) Substitute the gray parameter into the time function.

(10) $$x(t+1)=\left(x^{\left(0\right)}(1)-u/a\right)e^{-at}+u/a$$

(4) Reduce the derivative of $X^{\left(1\right)}$.

(11) $$x^{\left(0\right)}(t+1)=-a\left(x^{\left(0\right)}(1)-u/a\right)e^{-at}$$

(5) Calculate the absolute error and relative error of the residual.

(12) $$\left\{{}_{e(t)=\varepsilon^{\left(0\right)}(t)/x^{\left(0\right)}(t)}^{\varepsilon^{\left(1\right)}(t)=x^{\left(0\right)}(t)-x^{\left(0\right)}(t)x_{i}^{g+1}}\right.$$

(6) Perform posterior residual checks; first, calculate the observed data deviation $S_{1}$ and the deviation $S_{2}$ of the residuals.

(13) $$\left\{{}_{S_{2}{}^{2}=\frac{1}{m-1}\sum_{t=1}^{m-1}(q^{\left(0\right)}(t)-q^{-(0)}(t){)}^{2}}^{S_{1}{}^{2}=\sum_{t=1}^{m}(x^{\left(0\right)}(t)-x^{-(0)}(t){)}^{2}}\right.$$

(7) Calculate the variance ratio and the probability of small errors.

(14) $$\left\{{}_{P=\left\{\left|q^{\left(0\right)}(t)-q^{-(0)}\right|<0.6754S_{1}\right\}}^{c=S_{2}/S_{1}}\right.$$

The model is assessed according to the posterior ratio c and the small error probability *P*. When *P* > 0.95 and *c* < 0.35, the model is considered to be reliable, and a prediction can be made for that time; otherwise, Formula (10) must be corrected by analysis of the residual series.

#### 4.2.3. Multivariate Linear Programing Model

1. Land use carbon emissions

The optimization objective of this function was to minimize the net final carbon emissions of different land use types. Six land use type variables (forest land ($x_{1}$), grassland ($x_{2}$), cultivated land ($x_{3}$), water ($x_{4}$), construction land ($x_{5}$) and bare land ($x_{6}$)) were used, and the objective function for the final land use carbon emissions was

(15) $$f_{1}(x)=\sum_{i=1}^{6}k_{i}\times S_{i}\to \min$$

where $f_{1}(x)\to \min$ indicates that the net land use carbon emissions tend toward minimal values. The carbon emission factor of construction land in the target year is predicted by the GM(1,1) model(Table 2).

2. Land use economic efficiency

Land directly generates economic benefits and promotes economic development by supporting economic exchange and the development of economic activity [41]. It is therefore necessary to control land use carbon emissions while maximizing the economic benefits of land use. Using the methods described in previous studies, we obtained the economic efficiency coefficients of each land use type in each year from 2000 to 2020 by dividing the total economic output of the industries corresponding to each land use type in each year by the GDP for the year and fitting a linear regression equation. The economic efficiency coefficient of each land use type in the target year was expressed as a percentage of the annual total. The objective function for the economic efficiency of land use was

(16) $$f_{2}(x)=\sum_{i=1}^{6}e_{i}\times S_{i}\to \max$$

where $f_{2}(x)\to \max$ indicates that the economic efficiency of land use tends toward maximum values, and $e_{i}$ is the economic efficiency coefficient representing the unit economic output of land type *i*.

3. Land use eco-efficiency

We used an economic assessment of the value of environmental services to create the ecological benefit function. Our assessment was based on the research results of Xie G. D. et al. [42], and the environmental services values per unit area for different ecosystems were modified to reflect the observed situation in Sanmenxia. The eco-efficiency coefficients of different land use types were combined in the land use eco-efficiency objective function:

(17) $$f_{3}(x)=\sum_{i=1}^{6}p_{i}\times S_{i}\to \max$$

where $f_{3}(x)\to \max$ indicates that the ecological benefits of land use tend toward maximum values, and $p_{i}$ is the eco-efficiency coefficient representing the ecological benefits in terms of the unit economic output of land use type *i*.

4. Comprehensive objective function

The carbon emission function, economic benefit function and ecological benefit function were weighted and combined to form a comprehensive optimization equation. The respective weights were 0.5, 0.4 and 0.1 and were derived from existing research [25,43]. The three objective functions have different orders of magnitude and different units, so the comprehensive objective function must be dimensionless. The planned values of the three objective functions in 2030 were, respectively, 59,702.47 Mt, CNY 2642.26 million and CNY 31,546.20 million according to the *Sanmenxia City Territorial Spatial Plan*. The final comprehensive objective function was

(18) $$F(x)=-0.5\times \frac{f_{1}(x)}{59702.47}+0.4\times \frac{f_{2}(x)}{2642.26}+0.1\times \frac{f_{{}_{3}}(x)}{31546.20}$$

The comprehensive objective function minimizes carbon emissions and maximizes economic and ecological benefits of land use in Sanmenxia. We represented the comprehensive objective function by a linear programing model:

(19) $$F(x)=-2038.88x_{1}-3866.24x_{2}+37682.93x_{3}-17082.84x_{4}+357367.38x_{5}-481.08x_{6}$$

Seven constraints imposed by the socio-economic and environmental conditions and characteristics of land use in Sanmenxia and the master plan control index, which consists of the documents *Sanmenxia City Territorial Spatial Plan*, *The Fourteenth Five-Year Plan for National Economic and Social Development of Sanmenxia City and the Vision 2035 and National Land Planni* *ng Outline 2016–2030*, were applied to the comprehensive objective function. The specific constraints are given as Equations (19)–(25) and are informed by the following considerations.

(1) Rapid economic development and urbanization in Sanmenxia during the past decade has increased construction land use and has led to a decrease in cultivated land and grassland use. It has therefore become necessary to increase the protection of cultivated land resources and strictly observe the red line around cultivated land. The national cultivated land reserve of at least 124,300 km^2^ in 2020 and at least 121,666 km^2^ in 2030 indicates that the annual reduction in cultivated land use should be controlled at 0.2%.
(2) Construction land use makes the greatest contribution to the GDP but is also the main source of carbon emissions. Population growth has caused a rapid expansion in construction land use in Sanmenxia, and as economic growth continues, the area of construction land use will continue to increase in the future but will be constrained by intensifying construction land use. We therefore take the as-is growth in the area of construction land use as the lower limit and the planned area as the upper limit.
(3) Forest land use has the highest carbon sequestration benefit, and the area of forest land use increased annually over the last decade. Future development should be based on key environmental projects, such as natural forest resource protections and returning cultivated land to forest and grassland, to ensure that the predicted area of forest land use cannot be less than the current area. In addition, the rate of reduction in grassland use cannot exceed the rate of reduction over the last ten years.
(4) Water and natural land uses have varied little in the last 10 years, so the land use area of water and natural land should be maintained within this range.

The preceding analysis led to the following constraints on the objective function:

(20) $$\sum_{i=1}^{6}x_{i}=\begin{array}{r} 9936.52 \end{array}$$

(21) $$x_{1}\geq 4302.86$$

(22) $$1366.96\leq x_{2}\leq 1599.49$$

(23) $$3374.43\leq x_{3}\leq 3607.57$$

(24) $$136.95\leq x_{4}\leq 169.72$$

(25) $$446.58\leq x_{5}\leq 486.83$$

(26) $$1.76\leq x_{6}\leq 4.65$$

### 4.3. Land Use Spatial Prediction Model

The recently developed PLUS model is based on metacellular automata. Its application of new strategies for the analysis of land use change gives a better explanation of the mechanisms of land use change than previous explanations [44]. The model combines rule mining using a land expansion analysis strategy (LEAS) with a metacellular automata model using several random patch seeds (CARS) to identify changes in land use over two periods and formalizes the relationships between land use change and its drivers using a random forest algorithm. LEAS was used to calculate the probability of growth for each land use type in the study area, and the spatial pattern of future land use was predicted using CARS by combining the image elements, transformation matrices and domain weights of different land use types.

**Figure 3:**
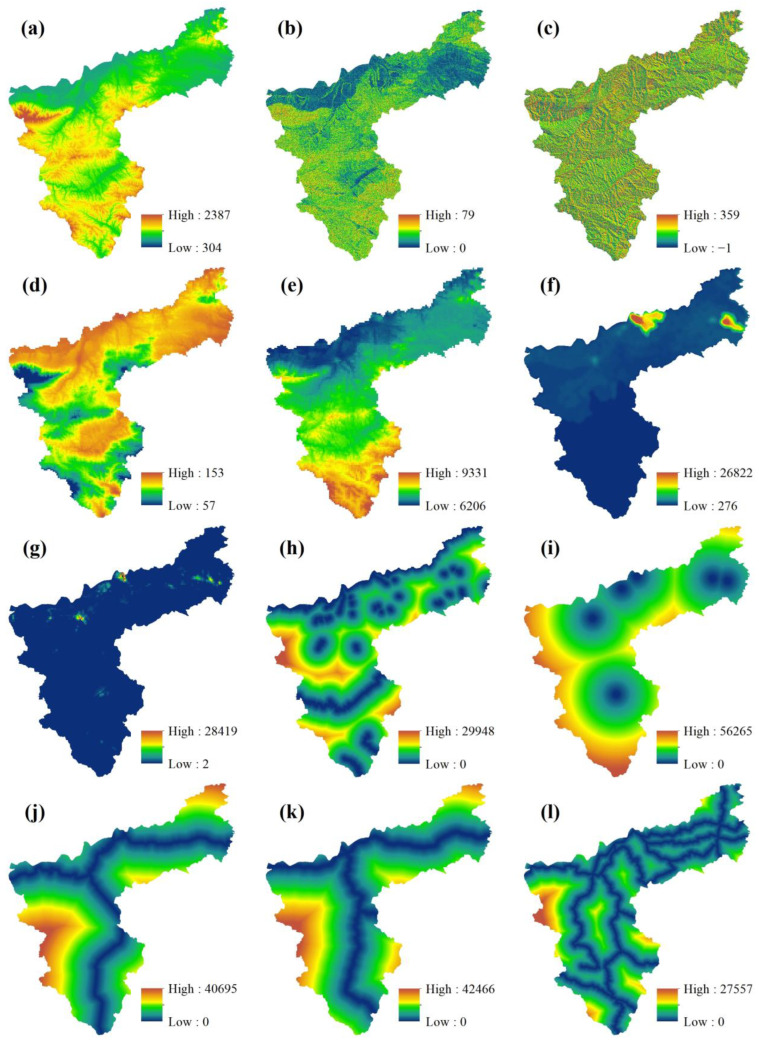
The main driving factors of land use change in Sanmenxia ((**a**), DEM; (**b**), slope; (**c**), aspect; (**d**), temperature; (**e**), amount of precipitation; (**f**), GDP; (**g**), population density; (**h**), distance to river; (**i**), distance to administrative center; (**j**), distance to railway; (**k**), distance to highways; (**l**), distance to road).

**Figure 4:**
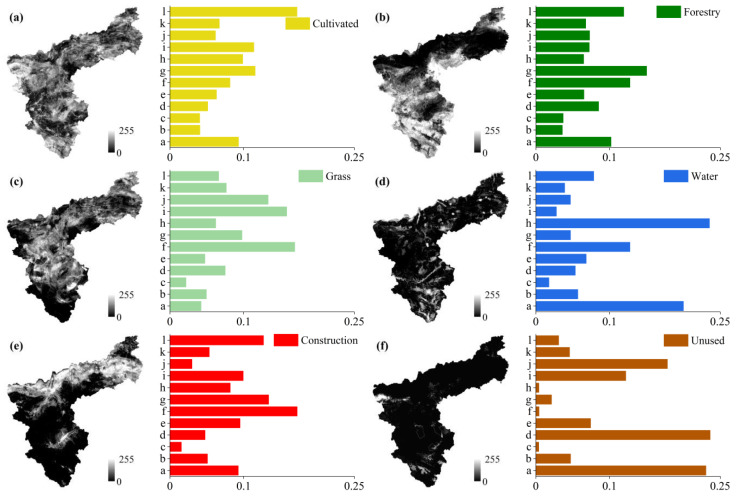
The weights of different drivers in influencing the change in land use ((**a**). band 1, cultivated; (**b**). band 2, forest; (**c**). band 3, grass; (**d**). band 4, water; (**e**). band 5, construction land; (**f**). band 6, unused).

**Figure 5:**
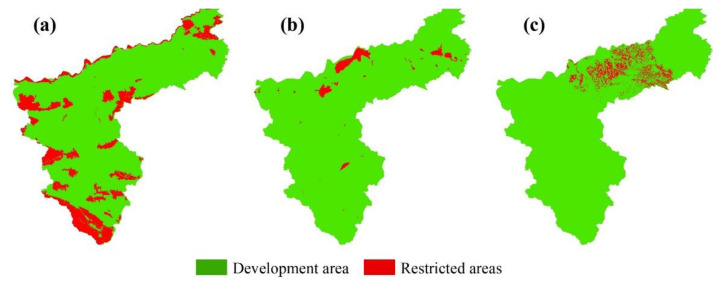
Restricted development areas ((**a**), ecological conservation area; (**b**), urban development boundaries; (**c**), permanent prime cultivated land).

(1) The PLUS model includes a Markov chain model, which is used to create a probability matrix of change in land use type and to predict land use for the as-is development scenario.
(2) Before and after the land use images for the two periods were input, LEAS was used to identify the areal growth in land use type between the two periods, and the data were sampled to identify the drivers (Figure 3). The drivers were quantified, and the probabilities of land use change and the degree of influence of each driver on each land use type were determined by random forest classification (RFC) (Figure 4). The drivers were selected to include the natural geographical characteristics of the region and socio-economic development factors. Taking into account the specific situation of Sanmenxia, 12 driving factors were identified and were processed using ArcGIS10.7 to ensure the same projection coordinates, spatial resolution and number of ranks.
(3) Finally, permanent prime cultivated land, areas within the ecological conservation area and areas within urban development boundaries were selected as restricted areas (Figure 5), which were binarized, with an unchangeable area being 0 and an area available for conversion being 1. The constrained future land use was predicted using a CA model with multi-class stochastic patch seeding (CARS). The 2020 land use image was predicted by inputting the images for 2000 and 2010 and using actual land use for 2020 as a quantitative constraint on the model. The kappa coefficient was obtained by comparing the actual vs. predicted values for 2020, and the kappa value 0.92 indicates that the prediction was good. The 2010 and 2020 land use images were therefore used as the base period images to predict the 2030 land use images.

## 5. Results

### 5.1. Spatial and Temporal Patterns of Land Use

**Figure 6:**
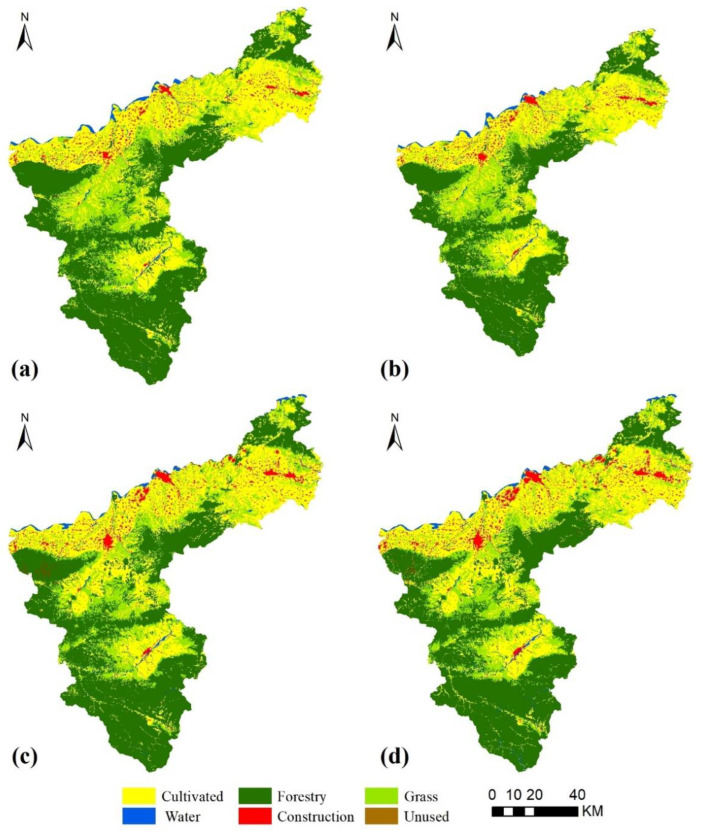
Spatial distribution of land use in Sanmenxia ((**a**), 1990; (**b**), 2000; (**c**), 2010; (**d**), 2020).

**Figure 7:**
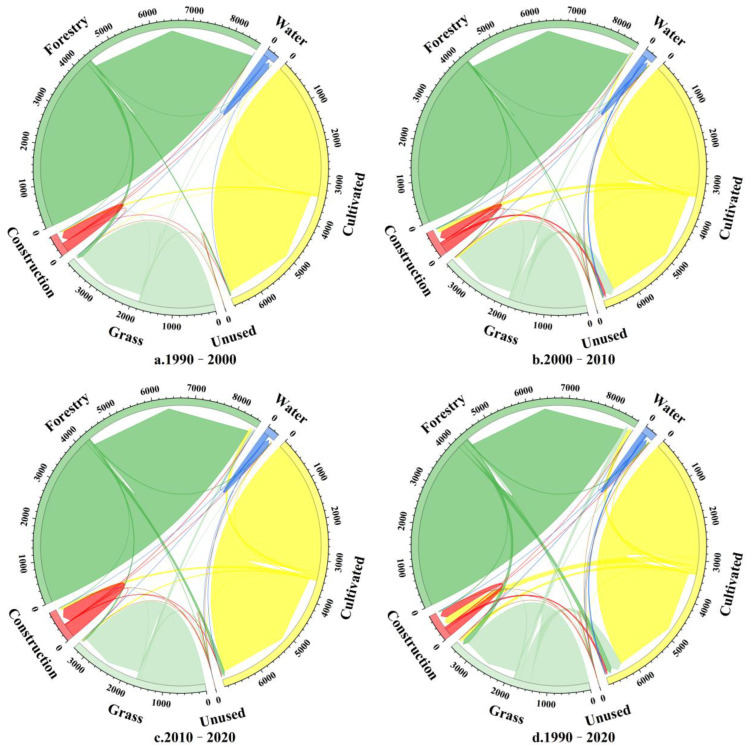
Variation in land use type during different periods.

Based on the land use images of Sanmenxia City from 1990 to 2020 (Figure 6), the area transfer matrix projections for different land use types were calculated (Figure 7). The land use types in Sanmenxia were mostly forest land, cultivated land and grassland. The total area of land use change from 1990 to 2020 was 1273.65 km^2^ and mainly involved cultivated land, forest land and grassland, with an overall increasing trend. From 1990 to 2000, forest land was converted to grassland and cultivated land (60.35%), and cultivated land was converted to construction land (38.46 km^2^), but this change in cultivated land was compensated for by the conversion of forest and grassland to cultivated land, which made the area of cultivated land relatively stable. From 2000 to 2010, the areas of grassland and cultivated land were mainly converted to other land use types, with an area share of 46.31% and 30.08%, respectively. There was a rapid expansion of construction land use, which increased by 102.20 km^2^ and was mainly converted from cultivated land, but the compensatory conversion of grassland to cultivated land maintained the general trend of cultivated land increasing in area. During 2010–2020, a balance between maintenance and conversion was reached between cultivated land and forest and grassland, although the area of cultivated land showed a decreasing trend as it continued to be converted into construction land, but the area of construction land decreased by 10.64% when compared to the previous period. In the 30-year period, the small areas of water and natural land use remained steady, but the areas of construction land and cultivated land use, which are the principal sources of carbon emissions, increased, respectively, by 189.43 km^2^ and 81.65 km^2^. These increases greatly diminished the areas of forest land and grassland use and seriously affected the total carbon sequestration in the study area.

### 5.2. Land Use Carbon Flow Analysis

Using the land use carbon calculation method, the carbon emissions (carbon sources minus carbon sinks) from 1990 to 2020 were calculated for the six land use types (Table 3). For the 1990–2020 period, the carbon emissions and carbon sinks for each land use type were stable, except that the carbon emissions from construction land as a whole, which initially increased and finally decreased, clearly show a growth trend and influenced the overall carbon emissions. Noticeably, from 2000 to 2010, the growth rate of total carbon emissions was at a maximum of 215.44%, but from 2010 to 2020, growth was negative, and carbon emissions decreased by 15.74%.

The carbon transfer between different land use types from 1990 to 2020 was calculated by the carbon flow model (Table 4). The table shows that the carbon flows in all three periods were positive, which indicates that the carbon emissions in the study area were continually increasing. In land use conversion, the main sources of negative carbon sinks were the carbon sinks resulting from the conversion of construction land to other land uses. The proportion of carbon due to conversion of construction land was 69.02% during 1990–2000 and was >95% during the two later periods, which indicates that the environmental restoration of mines in Sanmenxia had remarkable results; the number of green mines increased annually. The positive carbon flow showed a trend of first increasing and then decreasing from 1990 to 2020, but it still increased significantly overall. This increase was mainly due to increased carbon emissions from the conversion of other land uses to construction land use. Although this carbon flux was influenced by differences in carbon emission coefficients of construction land in different periods, it was primarily due to the conversion of cultivated land to construction land, which accounted for 40% of all land use conversion in 1990–2010 and 87.43% in 2010–2020. Although negative carbon flows increased annually, they were still far less than positive carbon flows, which indicates that there still exists the problem of increased positive carbon flows due to the massive increase in construction land use, and thus, the spatial pattern of land use was not optimal. This lack of optimality suggests that the carbon emission imbalance in the study area will continue to increase if the land use structure continues to develop according to the current patterns.

### 5.3. Predicting Spatial Pattern of Land Use

**Figure 8:**
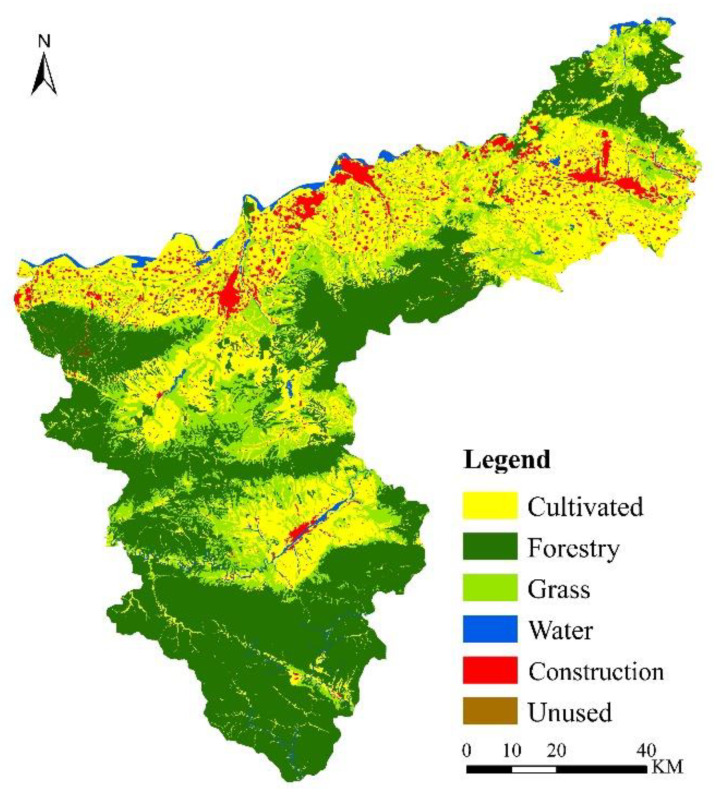
Land use pattern of as-is development for 2030.

The Markov chain method was used to predict land use distribution in 2030 based on the predicted land use structure in the as-is development scenario (Table 5). The area of cultivated land was predicted to decrease by 60.32 km^2^ and the area of grassland by 26.70 km^2^ over 2020 levels, while the area of construction land showed the greatest predicted increase of 58.17 km^2^, and forest land (10.23 km^2^), water (14.77 km^2^) and bare land (2.94 km^2^) all had small increases. The land use conversion matrix shows that the largest area of land use conversion in 2020–2030 was cultivated land (183.04 km^2^), followed by grassland (114.45 km^2^) and forest land (66.37 km^2^). Of these land use conversions, 37.87% of the lost cultivated land was converted to construction land, 31.13% to grassland and 20.08% to forest land; 63.04% of the lost grassland was converted into cultivated land and 29.89% into forest land; 90.39% of the increased area of construction land was from the conversion of cultivated land, and according to our projections using the PLUS model to obtain the land use in 2030 (Figure 8), most of the converted construction land will be formed by the original construction land expanding radially in all directions to occupy cultivated land, which will reduce the fragmentation and increase the connectedness of construction land. The loss of cultivated land use will be compensated for by the cultivation of grassland in the northern plains. Figure 7a–c show that the increase in construction land use occurs mainly in Daying town, with a concentration of construction land use in that region. Construction land use in Zhangwan township in the southwest of Sanmenxia will also spread toward Daying. Construction land use in Lingbao city also shows a continuing trend of extending upward along the northeastern rivers. The construction land use in Mianchi county borders that in Yima city and will continue to expand northward along the Hengmian highway.

Carbon emissions were projected based on the 2030 land use predictions. The total carbon emissions will increase by 38%, carbon sinks will remain almost unchanged, and carbon sources will increase by 34% compared to 2020. Although carbon emissions will show positive growth, the growth rate will decrease significantly compared to the 1990–2000 and 2000–2010 periods. The continuous growth in carbon emissions is directly related to the current as-is land use distribution in Sanmenxia, so there is an urgent need to optimize the spatial pattern of land use in order to meet the targets of the carbon peaking plan by 2030.

### 5.4. Optimization of Spatial Patterns of Land Use

The DE algorithm predicted land use structure after low carbon optimization (Table 3). A comparison of this predicted land use structure with that under as-is development showed that all land use areas decreased, except for the areas of forest land and cultivated land, which increased. The decrease in construction land use area was greatest, totaling 23.05 km^2^, which mainly resulted from forest land being converted to construction land, but the increase in forest land use area was greater, totaling 67.37 km^2^; the increase in cultivated land use area (10.28 km^2^) and decrease in grassland use area (11.63 km^2^) follow the same pattern. The land use transfer matrix shows that 90.08% of the increase in construction land area comes from cultivated land, and the increase in forest land area comes mainly from cultivated land and grassland. These land use changes result in carbon emissions being reduced by 20.43 t with a 43.33% lower growth rate compared to the as-is development scenario. Therefore, in order to control carbon emissions through changes in land use, we must increase the forest cover in the study area and at the same time control the increase in construction land use.

**Figure 9:**
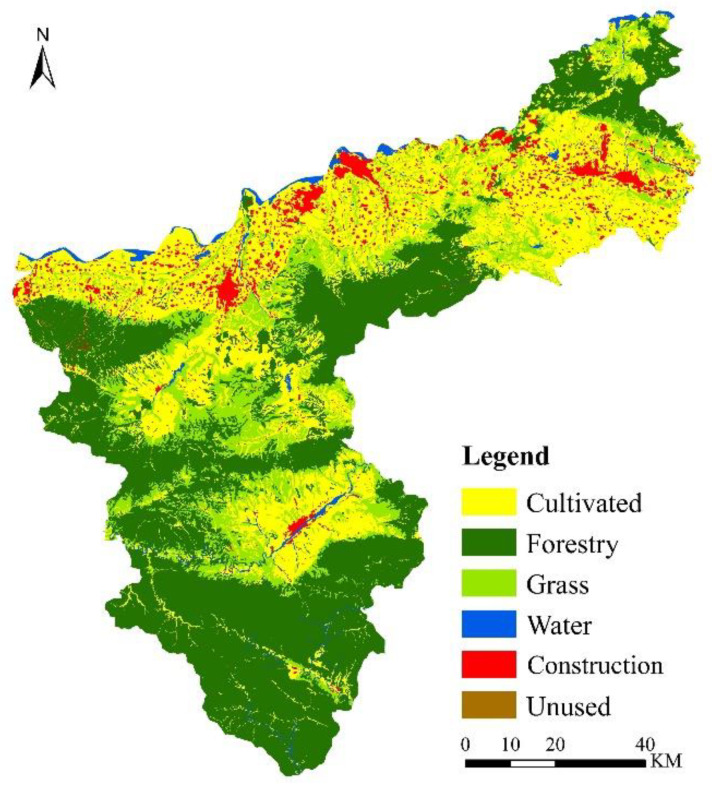
Spatial pattern of optimized land use for 2030.

**Figure 10:**
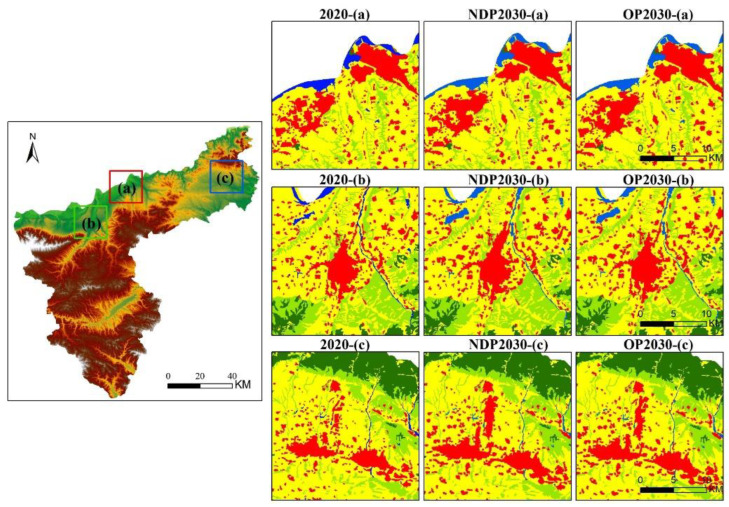
Comparison of land use in three regions under three scenarios: 2020, NDP2030 and OP2030 (2020 is land use in 2020; NDP2030 is land use in 2030 for as-is development; OP2030 is the spatial pattern of optimized land use for 2030).

The optimized low carbon land use structure was incorporated into the PLUS model to spatially optimize land use in Sanmenxia in 2030 (Figure 9). The results are consistent with the as-is predicted results before optimization, but the percentage in-crease in construction land use was lower and more concentrated, mainly in the northern urban area of Daying (area a in Figure 10) and in Lingbao along the northern river (area b in Figure 10) and in Mianchi along the border with Yima(area c in Figure 10). These changes are consistent with the direction of planned development in the *Sanmenxia City Master Plan (2013–2030)*.

## 6. Discussion

Socio-economic development, the greenhouse effect and global warming are becoming increasingly serious, and there is international consensus to control carbon emission in response to the goal of a carbon peak by 2030. Our analysis of the effects of carbon emissions due to land use changes in Sanmenxia from 1990 to 2020 and prediction of future change trends leads us to propose an optimization of the spatial distribution of land use constrained by minimal carbon production. Many studies have calculated land use carbon emissions using land use carbon emission coefficients and have analyzed the patterns and characteristics of carbon emissions to address the problems associated with land use patterns. We give more practical suggestions in terms of land use carbon emissions than previous studies and propose a spatial pattern of land use to minimize carbon production based on our analysis using a differential evolutionary algorithm combined with a PLUS model.

We selected carbon emission factors based on an analysis of emissions in the study area. For example, total end-use energy consumption in Sanmenxia was considered in the selection of carbon emission factors for areas of construction land use, and default emission factors were used to calculate carbon emissions from the consumption of raw coal, crude oil, gasoline and nine other energy sources in construction land use areas. This method is more accurate than using national averages. However, in the carbon flux calculations, we ignored ecosystem changes in carbon storage and considered only natural and anthropogenic carbon emissions; this approach may lead to discrepancies between model-predicted carbon emissions and measured emissions. In addition, we categorized land use types into six categories (cultivated land, forest land, grassland, water, construction land and unused land), which meant that we ignored the differences in carbon emissions that would be found in a more finely grained categorization; this inevitably led to some errors in the spatial distribution of carbon emissions.

In optimizing the land use structure, we maximized economic and ecological benefits and minimized carbon emissions to ensure that the economy and ecology of the study area were developed and protected while carbon emissions were controlled. In optimizing the 2030 objective function, we used a GM(1,1) model to predict the 2030 land use carbon emission coefficients instead of using the carbon emission coefficients of previous years. The calculation of the economic and ecological objective function required using linear fitting and other techniques to predict the correlation coefficients for 2030 before specifying the objective function. This approach ensured that the comprehensive objective function to optimize land use structure and the associated predicted carbon emissions should be close to the actual 2030 emissions. The spatial distribution was predicted using the PLUS model. Natural, social and economic factors were recognized as land use drivers, and the effects of future development planned for Sanmenxia were also considered, as were the ecological conservation area, changes in town boundaries and the recognition of a basis of permanent cultivated land, which were incorporated in the model as restricted areas.

These actions made the model more realistic and increased its practical value. The optimized predictions for 2030 show that it is necessary to strictly control land development to ensure a steady realization of economic and environmental benefits. In addition to controlling the increase in the area of construction land use, it is also necessary to take account of axial development and the geographical patterns of land use and thus expedite the creation of a belt-shaped town cluster of various land uses around the urban development space of the Shaanling Basin and the surrounding areas of the Longhai Railway, the Lianhuo Expressway and the National Highway 209–310. At the same time, the Mianchi–Yima industrial development wing must be constructed. These tasks will allow us to focus on land development in Sanmenxia and promote spatial intensification of land use in the limited land resource. Compared with some studies [10,21], this paper predicts the carbon emission coefficients in the target year when constructing the carbon emission function in the target year, and it also takes the economy and ecology into consideration, so that the obtained target function is more accurate. Meanwhile, the influence of future development planning policies is added in the land use simulation, and the obtained future spatial pattern of land use is closer to the real value.

## 7. Conclusions

In this study, we analyzed the carbon emission patterns of land use change in Sanmenxia city from 1990 to 2020 and constructed the spatial pattern of land use in 2030 under a low carbon orientation. The specific conclusions are as follows.

(1) From 1990 to 2020, the change in land use patterns in Sanmenxia city is obvious, mainly occurring as a conversion between cultivated land, forest land, grassland and construction land. Construction land use increased year by year, with 90.39% of the increased area coming from the conversion of cultivated land, and the areas of forest land and cultivated land generally tended to be stable, compensated mainly by the transformation of grassland. In the process of land use change, carbon source emissions first increased and then decreased due to the carbon emissions of construction land, but overall, the source far exceeded the carbon sink, and the carbon metabolism of land use was far from being in equilibrium.
(2) Carbon emissions from land use showed negative growth between 2010 and 2020. This phenomenon was influenced by a decrease in the carbon emission coefficient of construction land use over the period, but it was also related to the beginning of a decrease in the area of cultivated land use and a sudden increase in forest land use area in the period. These changes indicate that the change in carbon flow due to land use change is closely related to the change in land use patterns. This also indicates that the change in the patterns of energy consumption in Sanmenxia during the period led to a decrease in carbon emissions when compared with the previous period.
(3) The predictions for the as-is development scenario in Sanmenxia in 2030 show that the areas of cultivated land and grassland will decrease, respectively, by 60.32 km^2^ and 26.70 km^2^ over the previous period, and the area of construction land use will increase by 58.17 km^2^; the rate of increase in carbon emissions will reach 37.53%. A comparison between the low carbon optimized land use pattern and the land use pattern of the as-is development scenario shows that the increase in the area of construction land use will have an upper limit in 2030 and that the area of forest land use will continue to increase steadily; however, the total carbon emissions will decrease by 20.43 t, with a −43.33% negative growth rate. Newly converted construction land in the optimized land use pattern will be concentrated in the ribbon-cluster towns formed by the expansion around the Shaanling Basin along the Yellow River and in the Mianchi–Yima industrial development wing. This growth promotes the development and effective allocation of land resources in Sanmenxia.

## Figures and Tables

**Table 1:** Details of all data.

Data Type	Data Name	Time	Source
Raster data	Land Use Data (30 m)	2000, 2010, 2020	https://www.resdc.cn/ (accessed on 20 September 2022)
	DEM, Slope, Aspect	2020	https://urs.earthdata.nasa.gov/ (accessed on 20 September 2022)
	GDP	2019	https://www.resdc.cn/ (accessed on 20 September 2022)
	POP	2020	https://hub.worldpop.org/ (accessed on 20 September 2022)
	Meteorological Data	2020	http://data.cma.cn/ (accessed on 20 September 2022)
Vector data	Road Data	2020	https://www.webmap.cn/ (accessed on 20 September 2022)
	River system data	2020	https://data.casearth.cn/ (accessed on 20 September 2022)
Statistical data	Energy consumption; agricultural materials	2000–2020	Sanmenxia City Statistical Yearbook

**Table 2:** Projected land use carbon emission factor in Sanmenxia city in 2030.

Land Use Type	Predicted Results	*p*	c
Construction land	86.91	0.97	0.23

**Table 3:** Carbon emissions from different land use types from 1990 to 2020 (Mt).

Years	Cultivated	Forestry	Grass	Water	Construction	Unused	Carbon Source	Carbon Sink	Total
1990	14.79	−29.05	−0.39	−0.37	80.65	0.00	95.44	−29.81	65.63
2000	14.80	−28.26	−0.40	−0.39	121.69	0.00	136.49	−29.05	107.44
2010	15.42	−28.47	−0.35	−0.36	352.66	0.00	368.08	−29.18	338.90
2020	15.14	−28.57	−0.34	−0.40	299.73	0.00	314.87	−29.31	285.56

**Table 4:** Carbon flow from 1990 to 2020.

Direction of Carbon Flow	1990–2000 (t/y C)	2000–2010 (t/y C)	2010–2020 (t/y C)
Cultivated to Grass	−68.05	−2215.93	−1125.76
Construction land to Grass	−131.05	−6896.42	−5546.44
Forestry to Grass	6054.24	849.82	2134.01
Unused to Grass	0.00	0.00	−0.02
Water to Grass	0.67	66.46	9.94
Grass to Cultivated	1206.68	10,483.26	942.74
Construction land to Cultivated	−1512.04	−23,7481.24	−84,839.82
Forestry to Cultivated	2676.53	2329.44	8605.43
Unused to Cultivated	0.20	0.72	0.28
Water to Cultivated	8.13	2095.80	173.33
Grass to Construction land	1526.51	151,279.05	34,766.43
Cultivated to Construction land	167,234.65	1,022,698.96	469,174.72
Construction land to Construction land	239,651.08	1,238,949.28	−929,722.92
Forestry to Construction land	2748.60	122,516.66	18,975.47
Unused to Construction land	0.00	14,383.70	26.29
Water to Construction land	186.85	11,589.76	1160.80
Grass to Forestry	−270.72	−3049.29	−3867.51
Cultivated to Forestry	−64.90	−3307.81	−8525.20
Construction land to Forestry	−37.27	−4445.56	−33,036.17
Unused to Forestry	0.00	0.00	0.00
Water to Forestry	−1.37	−91.92	−104.53
Grass to Unused	0.15	0.00	0.33
Cultivated to Unused	0.00	−0.04	−0.12
Construction land to Unused	0.00	−3.95	−615.17
Forestry to Unused	0.00	0.00	177.34
Water to Unused	0.00	43.26	0.74
Grass to Water	−46.54	−138.02	−18.19
Cultivated to Water	−316.50	−802.02	−549.50
Construction land to Water	−30.69	−4333.02	−1958.88
Forestry to Water	22.71	128.84	506.69
Unused to Water	0.00	−75.49	−0.13
Positive carbon flow	765,668.55	2,930,380.67	887,674.17
Negative carbon flow	−2432.60	−257,226.84	−1,069,039.08
Total	763,235.95	2,673,153.83	−181,364.91

**Table 5:** Carbon emissions in 2030 for predicted and optimized land use patterns.

Land Use Type	Prediction for 2030		Optimization in 2030	
	Area (km^2^)	Carbon Emission (Mt)	Area (km^2^)	Carbon Emission (Mt)
Cultivated	3364.15	148,695.37	3374.43	149,149.88
Forestry	4313.80	−286,436.31	4381.17	−290,909.69
Grass	1611.12	−3383.35	1599.49	−3358.93
Water	171.05	−4327.44	169.72	−4293.88
Construction land	468.63	4,072,863.33	445.58	3,872,535.78
Unused	7.59	−3.79	1.76	−0.88

## Data Availability

The data used in this study are available from the first author upon reasonable request.
